# Comparison of Inflammatory Cytokine Levels in Hepatic and Jugular Veins of Patients with Cirrhosis

**DOI:** 10.1155/2023/9930902

**Published:** 2023-11-29

**Authors:** Leonard Kaps, Carolina Medina-Montano, Matthias Bros, Stephan Grabbe, Simon Johannes Gairing, Eva M. Schleicher, Stephan Gehring, Jörn M. Schattenberg, Peter R. Galle, Marcus-Alexander Wörns, Michael Nagel, Christian Labenz

**Affiliations:** ^1^Department of Internal Medicine I, University Medical Centre of the Johannes Gutenberg University, Mainz, Germany; ^2^Cirrhosis Centre Mainz (CCM), University Medical Centre of the Johannes Gutenberg University, Mainz, Germany; ^3^Department of Dermatology, University Medical Centre of the Johannes Gutenberg University, Mainz, Germany; ^4^Department of Paediatrics, University Medical Centre of the Johannes Gutenberg University, Mainz, Germany; ^5^Metabolic Liver Research Program, Department of Internal Medicine I, University Medical Centre of the Johannes Gutenberg University, Mainz, Germany; ^6^Department of Gastroenterology, Hematology, Oncology and Endocrinology, Klinikum Dortmund, Dortmund, Germany

## Abstract

**Background:**

Systemic inflammation with elevated inflammatory cytokines is a hallmark in patients with cirrhosis and the main driver of decompensation. There is insufficient data on whether inflammatory cytokine levels differ between hepatic and jugular veins, which may have implications for further immunological studies.

**Methods:**

Blood from the hepatic and jugular veins of 40 patients with cirrhosis was collected during hepatic venous pressure gradient (HVPG) measurements. Serum levels of 13 inflammatory cytokines (IL-1*β*, Int-*α*2, Int-*γ*, TNF-*α*, MCP-1, IL-6, IL-8, IL-10, IL-12p70, IL-17A, IL-18, IL-23, and IL-33) were quantified by cytometric bead array.

**Results:**

Cytokine levels of IFN-*α*2, IFN-*γ*, TNF-*α*, IL-6, IL-8, IL-10, IL-17A, IL-18, IL-23, and IL-33 were significantly elevated in patients with decompensated cirrhosis compared to patients with compensated cirrhosis. When comparing patients with clinically significant portal hypertension (CSPH, HVPG ≥ 10 mmHg) to patients without CSPH, there were significantly enhanced serum levels of IL-6 and IL-18 in the former group. There was no significant difference between cytokine serum levels between blood obtained from the jugular versus hepatic veins. Even in subgroup analyses stratified for an early cirrhosis stage (Child-Pugh (CP) A) or more decompensated stages (CP B/C), cytokine levels were similar.

**Conclusion:**

Cytokine levels increase with decompensation and increasing portal hypertension in patients with cirrhosis. There is no relevant difference in cytokine levels between hepatic and jugular blood in patients with cirrhosis.

## 1. Introduction

Cirrhosis is the end stage of chronic liver diseases and occurs in response to chronic liver injury [1]. In cirrhosis the liver's cellular architecture is severely disorganized due to an excessive amount of scare tissue, compromising its physiological function. Patients with cirrhosis have a high risk of life-threatening complications including variceal bleeding, hepatic encephalopathy (HE), or even acute-on-chronic liver failure (ACLF) [2, 3]. These complications are mainly triggered by increasing systemic inflammation due to inadequate immune response to pathogens caused by bacteria or bacterial components [4, 5].

Nonsterile systemic inflammation occurs when sinusoidal fibrosis impairs bacterial clearance by diminishing the expression of innate immune system proteins and pattern recognition receptors (e.g., toll-like receptors), which in turn reduces the bactericidal capacity of the liver [6]. By contrast, sterile inflammation primarily originates from translocation of bacterial products (e.g., lipopolysaccharides) from the gut to the portal circulation [7]. The gut's permeability increases because of portal hypertension mediated venous congestion in combination with dysbalanced microbiota composition towards pathogenic species [8, 9]. Both nonsterile and sterile systemic inflammation are associated with chronic activation of the innate and adaptive immune system resulting in broad production of inflammatory cytokines (e.g., tumor necrosis factor-*α* (TNF-*α*), interleukine-1 beta (IL-1*β*), IL-6 or interferon-*γ*) [4]. In advanced stages of cirrhosis, the immune system may even become exhausted under the constant inflammatory signaling, giving rise to an endotoxin tolerant state. An extreme outcome occurs in ACLF, where proinflammatory cytokines outbalance anti-inflammatory cytokines (e.g., IL-10) and a sepsis-like condition arises [10]. Besides their relevance for systemic inflammation, cytokines proved to be a valuable predictor for cirrhosis related complications. In this context, IL-6 was found to be predictive for the development of minimal and overt HE as well as mortality [11–13].

Although there is clear evidence for their prognostic and diagnostic relevance, the interplay between liver-derived and extrahepatic cytokines is complex as counter mechanisms in peripheral compartments may balance hepatic inflammatory stimuli. Currently, there is only insufficient data available on whether cytokine levels in the immediate vicinity of the liver, in the hepatic veins, differ from levels in the peripheral (e.g., jugular vein) circulation. Therefore, we aimed to quantify a set of inflammatory cytokines in the blood of close proximity to the liver (hepatic veins) and blood of liver distant vessels (jugular veins) of patients with compensated and decompensated cirrhosis.

## 2. Materials and Methods

In this retrospective study, the data of prospectively recruited patients (between 2020 and 2021) at the Cirrhosis Center Mainz (University Medical Center Mainz, Germany) were analyzed. All patients were recruited during elective measurement of the hepatic venous pressure gradient (HVPG). Diagnosis of liver cirrhosis was based on a combination of histology, conclusive appearance in ultrasound or radiological imaging, endoscopic features of portal hypertension, and medical history. No patients with any signs of active infection were included in the analyses.

Blood was obtained from all patients for biochemical analysis during HVPG measurement. In each patient, blood was obtained from the hepatic and the jugular vein. Blood from the hepatic vein was drawn with a blocked catheter in the hepatic vein.

Model of end-stage liver disease (MELD), Child-Pugh (CP), and CLIF-C AD score were used to estimate the severity of chronic liver disease [14–16]. Moreover, patients were classified according to the staging system published by D'Amico et al. [17]. Here, patients are grouped according to the presence of varices and the occurrence of decompensation events. In stage 1 and 2, patients with cirrhosis are compensated without or with varices, respectively. Stage 3 is characterized by variceal bleeding, while in stage 4 patients suffered from a first nonbleeding decompensating event. Stage 5 comprises all patients with any second decompensation event. According to the BAVENO VII definition, patients in stage 1 and 2 are compensated, while patients in higher stages 3, 4, and 5 are decompensated [17].

### 2.1. Healthy Controls

As a comparison to the cirrhosis group, blood from 40 healthy controls (age median 54 years (IQR 46; 60), 65% men) was analyzed. Blood was obtained from each patient from a cubital vein during an elective blood donation at the blood donation center of the University Medical Center Mainz. When volunteers register to become blood donors, they are regularly tested for infections of viral hepatitis B/C and human immunodeficiency virus. Furthermore, donors are not allowed to donate blood when they have symptoms of acute infection.

### 2.2. Hepatic Venous Pressure Gradient Measurement

HVPG was assessed according to a standardized protocol using an angled-tip balloon catheter under fluoroscopic control as described elsewhere [18]. The intake of nonselective *β*-blockers was interrupted for at least 5 days before HVPG measurement. Subclinical portal hypertension was defined by a HVPG of 6–9.5 mmHg and clinical significant portal hypertension (CSPH) by HVPG values ≥ 10 mmHg [19].

### 2.3. Quantification of Cytokine Levels by Cytometric Bead Array

After blood withdrawal, samples were incubated for 30 min to allow clotting and then centrifuged at room temperature at 2,000 rotations per minute (rpm) for 10 min. Then, the serum supernatant was transferred into 1.5 ml tubes and immediately stored at −80°C until further processing. On the day of analysis, diluted sera were thawed and cytokine concentrations for IL-1*β*, interferon (IFN)-*α*2, IFN-*γ*, TNF-*α*, monocyte chemoattractant protein (MCP)-1, IL-6, IL-8, IL-10, IL-12p70, IL-17A, IL-18, IL-23, and IL-33 were determined using the LEGENDplex™ Human Inflammation Panel 1 (13-plex) as recommended by the manufacturer's protocol instructions (Biolegend) [20]. In brief, samples were mixed with cytokine-specific capture beads and subsequently incubated with detection antibodies and then with PE-conjugated detection antibodies (all at room temperature in the dark under shaking) and subjected to flow cytometric analysis. Results were analyzed using Qognit Legendplex Analysis Software (Bio-legend, San Diego, CA). The minimum detectable concentration of cytokines in serum are: 1.5 + 0.6 (pg/ml) for IL-1*β*, 2.1 + 0.2 (pg/ml) for IFN-*α*2, 1.3 + 1.0 (pg/ml) for IFN-*γ*, 0.9 + 0.8 (pg/ml) for TNF-*α*, 1.1 + 1.2 (pg/ml) for MCP-1, 1.5 + 0.7 (pg/ml) for IL-6, 2.0 + 0.5 (pg/ml) for IL-8, 2.0 + 0.5 (pg/ml) for IL-10, 2.0 + 0.2 (pg/ml) for IL-12p70, 0.5 + 0.1 (pg/ml) for IL-17 A, 1.3 + 0.9 (pg/ml) for IL-18, 1.8 + 0.1 (pg/ml) for IL-23, and 4.4 + 1.5 (pg/ml) for IL-33.

### 2.4. Ethics

The study was conducted in accordance with the ethical guidelines of the 1975 Declaration of Helsinki (6th revision, 2008) and was approved by the ethics committee of the Landesärztekammer Rheinland-Pfalz (Nr. 837.052.12 (8153)). Written informed consent was obtained from all participants.

### 2.5. Statistics

Quantitative data are expressed as medians with interquartile ranges (IQR) or indicated otherwise. Categorical variables are given as frequencies and percentages. The Mann–Whitney *U*-test was used to test for any differences in metric variables. Differences between three or more groups with metric data were evaluated using an ordinary one-way ANOVA with Tukey's multiple comparisons test. Correlation analyses were conducted using Spearman's rank correlation. Our complete data analysis was exploratory. Hence, no adjustments for multiple testing were performed. For all tests, we used a 0.05 level to define statistically relevant deviations from the respective null hypothesis. However, due to the large number of tests, the *p*-values should be interpreted with caution and only as descriptive. Data of cytokine levels from healthy controls were excluded from group comparison when more than 50% of the samples were below the detection limit. Data were analyzed using GraphPad Prism Version 8.0.2 (GraphPad Software, San Diego, CA, USA).

## 3. Results

### 3.1. Baseline Characteristics

In total, data of 40 patients with cirrhosis were analyzed in this study. The majority of patients were men (65%) and the median age of the cohort was 59.5 years (IQR 51.7; 66.7). The etiology of cirrhosis was predominantly alcoholic (63%), followed by nonalcoholic fatty liver disease (27%), and a combination of viral hepatitis and alcoholic (10%). Most patients were categorized as CP stage A (47%) and B (45%), while only 3 (8%) were CP C. The median MELD score was 13 (9; 17.8). 12 (30%) patients were in a compensated stage of cirrhosis (D'Amico stage 1 and 2), while 28 (70%) were in a decompensated stage (D'Amico stage > 3). Of the 16 patients in D'Amico stage 5, three had ascites and variceal hemorrhage, the others had further decompensations with ascites. CSPH was present in 75% (*n* = 34) of the cohort. Additional characteristics of the cohort are displayed in Table 1. In addition to the cirrhosis cohort, cubital blood from 40 healthy controls was analyzed (age median 54 years (IQR 46; 60), 65% men). Standard laboratory parameters and reference cytokine levels of the healthy controls are shown in Table S1.

### 3.2. Comparison of Cytokine Serum Levels in Blood Obtained from Jugular and Hepatic Veins

**Figure 1:**
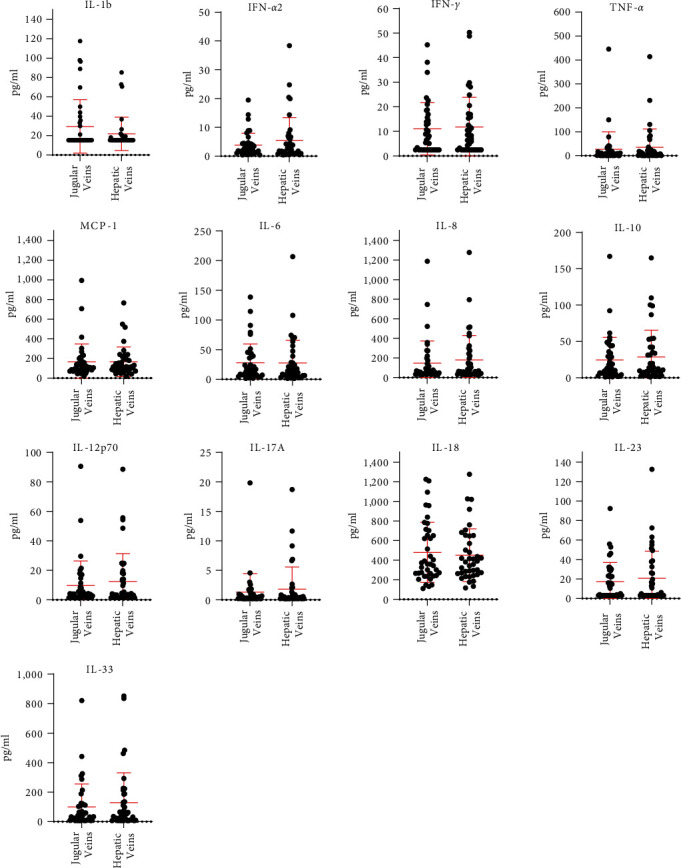
Comparison of serum cytokine levels between blood from hepatic and jugular veins. Data are expressed as individual data with mean plus standard deviation. Cytokine levels were determined as recommended by the manufacturer of the cytokine quantification kit [20]. IL, interleukin; IFN, interferon; MCP-1, monocyte chemotactic protein-1; TNF-*α*, tumor necrosis factor-*α*.

Cytokine serum levels determined in blood derived from hepatic and jugular veins of patients with cirrhosis are displayed in Figure 1. No relevant differences were detected between both sites of blood collection (*p* > 0.05 for each cytokine). For subgroup analyses, we separated the total cohort into subgroups according to CP A versus B/C as well as to compensated versus decompensated cirrhosis. Again, there were no relevant differences between cytokine serum levels between hepatic or jugular blood (*p* > 0.05 each) (Tables S2 and S3).

### 3.3. Analyses of Serum Cytokine Levels in Patients with Different Stages of Portal Hypertension, Decompensated Stages of Cirrhosis, and Alcoholic Cirrhosis

Patients with CSPH had significantly higher serum levels of IL-6 and IL-18 serum levels compared to patients without CSPH, both in hepatic as well as jugular blood (Table 2). IL-8 serum levels were only significantly higher in blood from hepatic veins of patients with CSPH compared to patients without CPSH (Table 2). CRP levels did not differ between the subgroups (Table S4). In the second step, patients were stratified according to a compensated (D'Amico stages 1 and 2, *n* = 12) or decompensated (D'Amico stages 3 and 4, *n* = 28) stage of cirrhosis. Here, patients with decompensated cirrhosis had significantly higher serum levels of Int-*α*2, Int-*γ*, TNF-*α*, IL-6, IL-8, IL-10, IL-23, and IL-33 compared to compensated patients, both in blood obtained from hepatic and jugular veins (Table 3). In addition, CRP levels were significantly higher in decompensated patients (Table S5). Moreover, IL-18 and IL-17A serum levels differed between both groups only in blood obtained from hepatic veins, while IL-23 serum levels did only differ between both groups in blood obtained from the hepatic veins (Table 3). Patients stratified for alcoholic versus nonalcoholic cirrhosis had similar cytokine levels both in blood obtained from hepatic and jugular veins (Figure S1).

### 3.4. Correlations between Serum Cytokine Levels and Demographic and Clinical Characteristics

**Figure 2:**
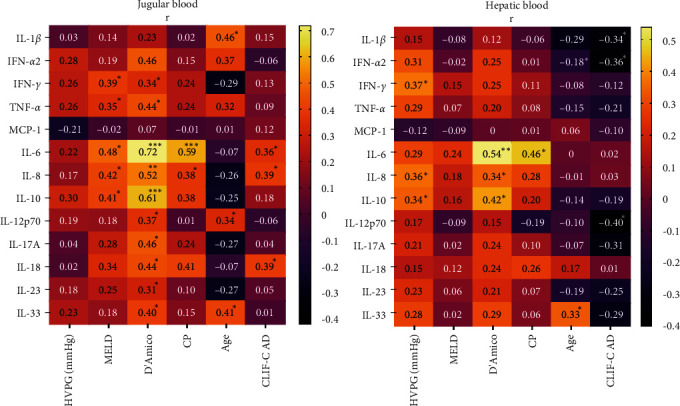
Correlations of serum cytokine levels in jugular and hepatic blood with clinical parameters shown as heat maps. *r* and *p* values ( ^*∗*^*p* < 0.05,  ^*∗∗*^*p* < 0.001,  ^*∗∗∗*^*p* < 0.0001) were calculated with Spearman correlations. CP, Child-Pugh; CLIF-C AD, chronic liver failure–consortium acute decompensation; HVPG, hepatic venous pressure gradient; IL, interleukin; IFN, interferon; MCP-1, monocyte chemotactic protein-1; MELD, model for end-stage liver disease; TNF, tumor necrosis factor.

Levels of inflammatory cytokines in hepatic and jugular veins were correlated with clinical parameters related to portal hypertension, liver function, and patients' characteristics. Correlations of cytokine levels were found for D'Amico stages, liver function (MELD and CLIF-C AD), and age. Regarding cytokines, IL-6 showed manifold correlations with MELD (hepatic veins, *r* = 0.48, *p*=0.002), CLIF-C AD (jugular veins, *r* = 0.36, *p*=0.02), D'Amico stages (jugular and hepatic veins, *r* = 0.72, *p* < 0.0001 and *r* = 0.54, *p*=0.003), and CP (jugular and hepatic veins, *r* = 0.59, *p* < 0.0001 and *r* = 0.46, *p*=0.003, Figure 2).

## 4. Discussion

In the present study, we were able to demonstrate that levels of cytokines, reflecting systemic inflammatory processes, are significantly increased in patients with decompensated cirrhosis or CSPH compared to patients with compensated cirrhosis or without CSPH. Moreover, we found no relevant difference of various serum cytokine levels in blood obtained from jugular or hepatic veins in patients with cirrhosis.

Systemic inflammation is a hallmark of the progression of liver cirrhosis [5]. Inflammatory cytokines and chemokines (a family of small cytokines) are thought to be major drivers of this phenomenon, but little is known about their origin and even less of their biodistribution [5, 21, 22]. In vitro studies have shown that cells of the innate immune system (primarily liver resident macrophages), hepatic stellate cells, and hepatocytes express inflammatory cytokines and chemokines during liver stress and injury [23, 24]. But also circulating immune cells might contribute to elevated levels of inflammatory cytokines in chronic liver disease [25, 26]. In this context, our study expands the existing literature by demonstrating that there is no relevant difference of the quantified inflammatory cytokines between blood obtained from jugular and hepatic veins in patients with cirrhosis. Similar results were also found in a recently published study investigating inflammatory as well as coagulation parameters in blood obtained from the portal vein, hepatic veins, and peripheral veins [27]. In our current study, the lack of differences in systemic inflammation between jugular and hepatic blood was also consistent in sensitivity analyses when stratifying the cohort for different disease stages or the presence of CSPH. As a consequence, the sampling of venous hepatic blood by invasive procedures such as HVPG measurement does not seem to be mandatory to assess levels of hepatic inflammatory cytokine for diagnostic or scientific purposes.

A plethora of cytokines with pleiotropic effects in systemic inflammation are emitted by cirrhotic livers [5, 21, 28–30]. We have selected a cytokine panel that encompasses several key inflammatory cytokines to delineate the degree of systemic inflammation in our patients (IL-1*β*, IFN-*α*2, IFN-*γ*, TNF-*α*, MCP-1, IL-6, IL-8, IL-10, IL-12p70, IL-17A, IL-18, IL-23, and IL-33). In the past, several studies investigated systemic inflammation in patients with cirrhosis and it is a well-accepted fact that bacterial translocation due to portal hypertension is a major driver of inflammatory processes [5]. Of note, bacterial translocation is not only associated with systemic inflammation but also several other alterations in the blood such as enhanced platelet activation, which may contribute to complications of cirrhosis, such as portal vein thrombosis [31]. In this context, it is of pivotal importance to develop treatment strategies to decrease systemic inflammation as a key driver of complications of cirrhosis to improve the long-term prognosis. One potential treatment combination that may be promising is rifaximin and simvastatin, which are currently being studied in a large trial [32]. However, it has to be mentioned that rifaximin had no major impact on the inflammatory state and only small effects on bacterial translocation in a preliminary study in patients with decompensated cirrhosis [33].

Our current study highlights the important role of IL-6 as a marker of impaired liver function by demonstrating significant correlations between higher IL-6 serum levels and clinical parameters such as MELD (*p*=0.002) D'Amico stages (*p* < 0.0001) and CP (*p* < 0.0001). Furthermore, IL-6 levels were significantly higher in patients with CSPH, which is one of the main drivers for life-threatening complications in cirrhosis such as bleeding of esophageal varices. It is a well-described fact that higher levels of inflammatory cytokines progress with the severity of cirrhosis and may trigger acute decompensation and also ACLF [5, 34]. IL-23 was significantly higher (hepatic blood, *p*=0.04) in patients with decompensated cirrhosis. This is an interesting finding because IL-23 can be produced by hepatic monocyte-derived macrophages, corroborating that myeloid cells fuel systemic inflammation in decompensated cirrhosis [21, 35, 36].

Our study has limitations that have to be acknowledged. First, due to our study design and the comparably small cohort, we are only able to identify potential associations and causality cannot be proven. Moreover, our sample size precludes adjustments for multiple testing. Therefore, our findings have to be strictly interpreted in the context of its design and as purely exploratory. Second, our study cohort is too small to conduct any analyses regarding hard clinical outcome events. Third, it would be interesting to investigate cytokine serum levels in jugular and hepatic veins of patients without cirrhosis, however, the invasive HVPG measurement would be unethical in these patients. Fourth, we did not test for bacteria in the sampled blood neither by microbiological nor bacterial DNA analysis. This may have ruled out clinical occult bacteremia, which could have influenced cytokine levels. Finally, we had no access to probes from the cubital vein in patients with cirrhosis and are therefore unable to analyze the biodistribution of the respective cytokines.

## 5. Conclusion

In conclusion, we were able to demonstrate that levels of cytokines, reflecting systemic inflammatory processes, are significantly increased in patients with decompensated cirrhosis or CSPH compared to patients with compensated cirrhosis or without CSPH. However, we found no relevant difference of various serum cytokine levels between blood obtained from jugular or hepatic veins in patients with cirrhosis. Whether sampling of hepatic blood can be completely avoided for immunological studies needs to be determined in follow-up studies.

## Figures and Tables

**Table 1:** Baseline characteristics of the study cohort.

Total patients, *n* (%)		40 (100%)
	Male gender, *n* (%)	26 (65%)
	Age (years), median (IQR)	59.5 (51.7; 66.7)

Etiology of cirrhosis	Alcohol, *n* (%)	25 (63%)
	NAFLD, *n* (%)	11 (27%)
	Mixed (viral hepatitis and alcohol), *n* (%)	4 (10%)

Liver function and portal hypertension	Child-Pugh score, *n* (%)	A 19 (47%), B 18 (45%), C 3 (8%)
	MELD, median (IQR)	13 (9; 17.8)
	D'Amico stages, *n* (%)	
	Stage 1	4 (10%)
	Stage 2	8 (20%)
	Stage 4	12 (30%)
	Stage 5	16 (40%)
	HVPG, mmHg (IQR)	17 (11; 20)
	HVPG stages, *n* (%)	
	6–9 mmHg	6 (15%)
	10–19 mmHg	22 (55%)
	≥20 mmHg	12 (30%)

Laboratory values, median	Sodium, mmol/l (IQR)	138 (135; 139)
	Potassium, mmol/l (IQR)	3.8 (3.6; 4.2)
	BUN, mg/dl (IQR)	14 (11; 21)
	SCr, mmol/l (IQR)	0.8 (0.7; 1.1)
	INR, median (IQR)	1.4 (1.2; 1.7)
	Bilirubin, mg/dl (IQR)	1.5 (1.3; 3.7)
	CRP, mg/l (IQR)	8.4 (4.5; 18)
	Albumin, g/l (IQR)	30 (24.5; 35)
	Thrombocytes, /nl (IQR)	110 (87; 150.5)
	WBC, /nl (IQR)	5.7 (4.2; 8.5)

*Note*: Data are expressed as medians with interquartile ranges or as frequencies with percentages; BUN, blood urea nitrogen; SCr, serum creatinine, HVPG, hepatic venous pressure gradient; MELD, model for end-stage liver disease; WBC, white blood cell count; INR, international normalized ratio; CRP, C-reactive protein.

**Table 2:** Comparison of serum cytokine levels between patients with and without CSPH in blood from jugular and hepatic veins.

	Jugular veins						*p*-Values
	Non-CSPH	IQR		CSPH	IQR		
IL-1*β*, pg/ml	15.3	15.3	24.0	15.3	15.3	40.7	0.40
Int-*α*2, pg/ml	1.8	1.1	4.3	2.6	1.3	4.4	0.49
Int-*γ*, pg/ml	5.9	2.5	9.2	9.7	2.5	19.3	0.26
TNF-*α*, pg/ml	2.9	1.0	13.4	10.0	1.2	22.0	0.26
MCP-1, pg/ml	107.4	104.4	138.2	110.9	70.4	195.3	0.73
IL-6, pg/ml	**7.2**	**4.4**	**11.4**	**18.1**	**8.2**	**43.9**	**0.03**
IL-8, pg/ml	40.0	32.8	56.2	69.1	35.5	202.9	0.08
IL-10, pg/ml (IQR)	6.7	2.4	13.7	14.7	6.0	43.8	0.13
IL-12p70, pg/ml	3.4	1.4	9.0	4.1	1.4	12.2	0.49
IL-17A, pg/ml	0.2	0.2	0.5	0.5	0.2	1.7	0.12
IL-18, pg/ml	**241.0**	**126.6**	**274.1**	**398.0**	**270.4**	**730.0**	**<0.01**
IL-23, pg/ml	4.5	3.2	19.7	11.7	3.2	29.5	0.73
IL-33, pg/ml	28.8	15.1	109.7	43.6	9.7	117.0	0.70

	Hepatic veins						

IL-1*β*, pg/ml	15.3	15.3	15.4	15.3	15.3	29.1	0.24
Int-*α*2, pg/ml	1.8	0.9	3.1	3.2	0.9	6.8	0.27
Int-*γ*, pg/ml	5.1	2.5	8.3	10.4	2.5	21.6	0.16
TNF-*α*, pg/ml	4.6	1.0	13.4	9.2	1.4	31.1	0.32
MCP-1, pg/ml	105.4	92.5	143.1	116.3	73.4	205.2	0.78
IL-6, pg/ml	**6.0**	**3.1**	**9.0**	**17.5**	**7.8**	**41.2**	**0.02**
IL-8, pg/ml	**32.8**	**32.8**	**61.0**	**85.5**	**37.3**	**281.9**	**0.02**
IL-10, pg/ml	8.0	2.7	12.0	14.0	6.7	44.9	0.13
IL-12p70, pg/ml	3.8	1.5	7.6	4.0	1.4	18.7	0.73
IL-17A, pg/ml	0.2	0.2	0.4	0.5	0.2	1.5	0.06
IL-18, pg/ml	**209.2**	**128.8**	**271.1**	**417.7**	**289.8**	**661.4**	**<0.001**
IL-23, pg/ml	3.6	3.2	11.9	9.6	3.2	49.8	0.27
IL-33, pg/ml	28.4	17.3	72.1	62.1	9.9	195.0	0.43

*Note*. Data are expressed as median with interquartile ranges (IQR). *p*-values were assessed by the Mann–Whitney *U*-test. When significance between the groups was reached, values are printed in bold. CSPH, clinical significant portal hypertension; IL, interleukin; IFN, interferon; MCP-1, monocyte chemotactic protein-1; TNF, tumor necrosis factor; CRP, C-reactive protein.

**Table 3:** Comparison of serum cytokine levels between patients with compensated and decompensated cirrhosis.

	Jugular veins						*p*-Values
	Compensated	IQR		Decompensated	IQR		
IL-1*β*, pg/ml	15.3	15.3	19.7	15.3	15.3	48.5	0.39
IFN-*α*2, pg/ml	**1.5**	**0.8**	**2.6**	**3.5**	**1.5**	**6.1**	**0.02**
IFN-*γ*, pg/ml	**3.0**	**2.5**	**7.1**	**12.9**	**3.1**	**21.6**	**0.02**
TNF-*α*, pg/ml	**2.7**	**1.0**	**7.8**	**13.3**	**2.9**	**32.5**	**<0.01**
MCP-1, pg/ml	105.9	86.5	139.5	114.0	70.0	206.0	0.74
IL-6, pg/ml	**6.7**	**3.4**	**8.6**	**22.5**	**11.6**	**45.5**	**<0.01**
IL-8, pg/ml	**34.9**	**32.8**	**52.7**	**78.0**	**41.9**	**260.8**	**0.01**
IL-10, pg/ml	**3.9**	**1.9**	**8.4**	**20.4**	**8.3**	**44.2**	**<0.01**
IL-12p70, pg/ml	3.4	1.4	4.6	5.0	1.5	16.5	0.10
IL-17A, pg/ml	**0.2**	**0.2**	**0.3**	**0.8**	**0.2**	**1.7**	**0.02**
IL-18, pg/ml	**254.4**	**212.5**	**563.5**	**388.2**	**272.3**	**767.9**	**0.04**
IL-23, pg/ml	3.2	3.2	4.8	19.1	3.2	31.3	0.06
IL-33, pg/ml	**15.5**	**9.0**	**32.8**	**61.5**	**12.3**	**172.5**	**0.02**

	Hepatic veins						

IL-1*β*, pg/ml	15.3	15.3	15.6	15.3	15.3	34.3	0.33
IFN-*α*2, pg/ml	**1.3**	**0.7**	**3.3**	**3.9**	**1.3**	**6.9**	**0.06**
IFN-*γ*, pg/ml	**3.8**	**2.5**	**6.7**	**11.5**	**3.5**	**23.8**	**0.02**
TNF-*α*, pg/ml	**2.3**	**1.0**	**7.9**	**17.7**	**1.8**	**34.7**	**0.03**
MCP-1, pg/ml	105.4	81.7	174.4	116.3	76.2	232.6	0.69
IL-6, pg/ml	**4.3**	**2.3**	**7.6**	**20.7**	**11.7**	**45.5**	**<0.01**
IL-8, pg/ml	**37.5**	**32.8**	**63.2**	**114.2**	**38.0**	**293.8**	**0.02**
IL-10, pg/ml	**5.8**	**2.4**	**9.4**	**19.3**	**8.1**	**50.2**	**0.01**
IL-12p70, pg/ml	3.4	1.4	5.2	4.4	1.5	19.4	0.33
IL-17A, pg/ml	0.2	0.2	0.5	0.5	0.2	1.9	0.06
IL-18, pg/ml	279.5	231.5	482.1	414.4	293.9	675.5	0.08
IL-23, pg/ml	**3.2**	**3.2**	**5.6**	**18.2**	**3.2**	**50.9**	**0.04**
IL-33, pg/ml	**15.9**	**7.2**	**57.1**	**63.2**	**18.5**	**207.1**	**0.04**

*Note*. Data are expressed as median with interquartile ranges (IQR). *p*-values were assessed by the Mann–Whitney *U*-Test. When significance between the groups was reached, values are printed in bold. IL, interleukin; IFN, interferon; MCP-1, monocyte chemotactic protein-1; TNF, tumor necrosis factor; CRP, C-reactive protein.

## Data Availability

The datasets generated during and/or analyzed during the current study are not publicly available but are available from the corresponding author on reasonable request.

## Abbreviations

ACLF: Acute-on-chronic liver failure
AST: Aspartate transaminase
ALT: Alanine transaminase
BUN: Blood urea nitrogen
CP: Child-Pugh
CLIF-C AD: Chronic liver failure–consortium acute decompensation
CRP: C-reactive protein
CSPH: Clinical significant portal hypertension
HE: Hepatic encephalopathy
HIV: Human immunodeficiency virus
HVPG: Hepatic venous pressure gradient
IL: Interleukin
IFN: Interferon
MCP-1: Monocyte chemoattractant protein-1
MELD: Model of end-stage liver disease
Rpm: Rotations per minute
TNF-*α*: Tumor necrosis factor-*α*
WBC: Whole blood count interleukin.

## Supplementary Materials

Supplementary Materials
*Table S1*: laboratory parameters and cytokine levels of the healthy control cohort. *Table S2:* comparison of serum cytokine levels between blood from hepatic and jugular veins in patients with early stage cirrhosis (Child-Pugh stage A) and advanced stage cirrhosis (B and C). *Table S3*: comparison of serum cytokine levels between blood from hepatic and jugular veins in patients stratified by compensated versus decompensated cirrhosis. *Table S4*: comparison of C-reactive protein concentration between patients with and without CSPH in blood from jugular veins. *Table S5*: comparison of C-reactive protein concentration between patients with compensated and decompensated cirrhosis in blood from jugular veins. *Figure S1*: comparison of serum cytokine levels between blood from hepatic and jugular veins in patients with alcoholic versus nonalcoholic cirrhosis.
