# Supplementary material for: Comparison of Inflammatory Cytokine Levels in Hepatic and Jugular Veins of Patients with Cirrhosis
Source: Mediators Inflamm. 2023 Nov 29;2023:9930902. doi: 10.1155/2023/9930902 (PMC10700970; doi:10.1155/2023/9930902)
Supplement: Supplementary Materials — Table S1: laboratory parameters and cytokine levels of the healthy control cohort. Table S2: comparison of serum cytokine levels between blood from hepatic and jugular veins in patients with early stage cirrhosis (Child-Pugh stage A) and advanced stage cirrhosis (B and C). Table S3: comparison of serum cytokine levels between blood from hepatic and jugular veins in patients stratified by compensated versus decompensated cirrhosis. Table S4: comparison of C-reactive protein concentration between patients with and without CSPH in blood from jugular veins. Table S5: comparison of C-reactive protein concentration between patients with compensated and decompensated cirrhosis in blood from jugular veins. Figure S1: comparison of serum cytokine levels between blood from hepatic and jugular veins in patients with alcoholic versus nonalcoholic cirrhosis. [file 9930902.f1.docx]

**Supplementary data**

**Supplementary table 1.** Laboratory parameters and cytokine levels of the healthy control cohort

| **Standard laboratory (n=40)** | | **Cytokine levels (n=40, pg/ml)** | |
| --- | --- | --- | --- |
| **Sodium, mmol/l (IQR)** | 141 (139; 142) | **Il-1b** | BDL (<1.5 +/- 0.6) |
| **Potassium, mmol/l (IQR)** | 4.2 (4; 4.4) | **IFN-α2** | 2.4 (+/-1.4) |
| **SCr, mmol/l (IQR)** | 0.9 (0.7; 0.9) | **IFN-Υ** | BDL (< 1.3 +/- 1.0) |
| **BUN, mg/dl (IQR)** | 14 (11; 16) | **TNF-α** | BDL (< 0.9 +/- 0.8 ) |
| **ALT (U/l)** | 15 (12; 22) | **MCP-1** | 111.6 (+/- 95.4) |
| **AST (U/l)** | 28 (25; 31) | **IL-6** | 8.4 (+/- 21.9) |
| **Bilirubin, mg/dl (IQR)** | 0.4 (0.3; 0.4) | **IL-8** | 22.7 (+/- 31.7) |
| **CRP, mg/l (IQR)** | 1.1 (0.4; 2.4) | **IL-10** | BDL (<2.0 +/- 0.5) |
| **Albumin, g/l (IQR)** | 41 (39; 43) | **IL-12p70** | BDL (< 5 +/- 8.1) |
|  |  | **IL-17A** | 0.6 (+/- 2.3) |
|  |  | **IL-18** | 204 (+/- 86.1) |
|  |  | **IL-23** | BDL (< 1.8 +/- 0.1) |
|  |  | **IL-33** | BDL (< 4.4 +/- 1.5) |

Data are expressed as median with interquartile ranges (IQR) for the standard laboratory or average with standard deviation for the cytokine levels; Data was set as BDL when > 50% of the samples were below the detection limit. AST, Aspartate transaminase; ALT, Alanine transaminase; BDL, below detection limit; BUN, blood urea nitrogen; SCr, serum creatinine, CRP, C-reactive protein

**Supplementary table 2.** Comparison of serum cytokine levels between blood from hepatic and jugular veins in patients with early-stage cirrhosis (Child-Pugh stage A) and advanced stage cirrhosis (B and C).

|  | **CP A** | | | | | | **p-values** |
| --- | --- | --- | --- | --- | --- | --- | --- |
|  | **Jugular veins** | **IQR** | | **Hepatic veins** | **IQR** | |  |
| IL-1β, pg/ml | 15.3 | 15.3 | 39.7 | 15.3 | 15.3 | 26.5 | 0.6 |
| IFN-α2, pg/ml | 1.8 | 0.8 | 4.3 | 2.8 | 0.7 | 7.0 | 0.5 |
| IFN-γ, pg/ml | 5.4 | 2.5 | 12.7 | 5.9 | 2.5 | 24.8 | 0.5 |
| TNF-α, pg/ml | 4.4 | 1.0 | 11.1 | 6.5 | 1.0 | 67.7 | 0.4 |
| MCP-1, pg/ml | 106.2 | 70.8 | 199.8 | 106.6 | 77.1 | 220.1 | 0.8 |
| IL-6, pg/ml | 7.2 | 4.0 | 18.1 | 6.3 | 3.3 | 21.3 | 1 |
| IL-8, pg/ml | 39.8 | 32.8 | 70.0 | 59.9 | 32.8 | 88.0 | 0.4 |
| IL-10, pg/ml | 6.2 | 2.0 | 28.7 | 9.0 | 2.8 | 33.7 | 0.4 |
| IL-12p70, pg/ml | 3.8 | 1.4 | 8.5 | 4.5 | 2.5 | 18.3 | 0.4 |
| IL-17A, pg/ml | 0.2 | 0.2 | 1.0 | 0.4 | 0.2 | 0.7 | 0.5 |
| IL-18, pg/ml | 274.0 | 204.7 | 358.8 | 291.2 | 231.0 | 654.1 | 0.6 |
| IL-23, pg/ml | 3.9 | 3.2 | 29.3 | 4.1 | 3.2 | 49.3 | 0.6 |
| IL-33, pg/ml | 32.0 | 9.8 | 111.7 | 35.0 | 9.2 | 213.1 | 0.6 |
|  | **CP B and C** | | | | | |  |
|  |  |  |  |  |  |  |  |
| IL-1β, pg/ml | 15.3 | 15.3 | 41.9 | 15.3 | 15.3 | 21.6 | 0.6 |
| IFN-α2, pg/ml | 2.6 | 1.5 | 7.1 | 2.6 | 1.1 | 6.3 | 0.8 |
| IFN-γ, pg/ml | 13.5 | 5.2 | 21.6 | 10.9 | 3.5 | 16.8 | 0.5 |
| TNF-α, pg/ml | 13.3 | 5.1 | 31.3 | 12.5 | 1.8 | 24.1 | 0.6 |
| MCP-1, pg/ml | 110.9 | 73.3 | 163.4 | 116.3 | 77.0 | 157.5 | 1 |
| IL-6, pg/ml | 36.8 | 13.3 | 69.8 | 20.5 | 11.7 | 57.5 | 0.2 |
| IL-8, pg/ml | 97.3 | 41.9 | 275.9 | 114.2 | 36.7 | 238.7 | 0.9 |
| IL-10, pg/ml | 20.4 | 8.5 | 48.1 | 14.0 | 7.7 | 50.2 | 0.7 |
| IL-12p70, pg/ml | 4.2 | 1.5 | 13.9 | 3.4 | 1.4 | 13.3 | 0.6 |
| IL-17A, pg/ml | 0.6 | 0.2 | 1.8 | 0.4 | 0.2 | 1.3 | 0.6 |
| IL-18, pg/ml | 477.7 | 293.9 | 825.8 | 418.8 | 307.0 | 650.5 | 0.4 |
| IL-23, pg/ml | 19.1 | 3.2 | 29.8 | 14.8 | 3.2 | 37.1 | 1 |
| IL-33, pg/ml | 56.9 | 11.8 | 247.0 | 62.1 | 12.9 | 173.3 | 1 |

Data are expressed as median with interquartile ranges (IQR). P-values were assessed by Mann-Whitney-U-Test. IL, Interleukin; IFN, Interferon; MCP-1, Monocyte chemotactic protein-1; TNF, Tumor necrosis factor.

**Supplementary table 3.** Comparison of serum cytokine levels between blood from hepatic and jugular veins in patients stratified by compensated versus decompensated cirrhosis.

|  | **Compensated cirrhosis** | | | | | | **p-values** |
| --- | --- | --- | --- | --- | --- | --- | --- |
|  | **Jugular veins** | **IQR** | | **Hepatic veins** | **IQR** | |  |
| IL-1β, pg/ml (IQR) | 15.3 | 15.3 | 19.7 | 15.3 | 15.3 | 15.6 | 0.9 |
| IFN-α2, pg/ml (IQR) | 1.5 | 0.8 | 2.6 | 1.3 | 0.7 | 3.3 | 0.9 |
| IFN-γ, pg/ml (IQR) | 3.0 | 2.5 | 7.1 | 3.8 | 2.5 | 6.7 | 1 |
| TNF-α, pg/ml (IQR) | 2.7 | 1.0 | 7.8 | 2.3 | 1.0 | 7.9 | 0.9 |
| MCP-1, pg/ml (IQR) | 105.9 | 86.5 | 139.5 | 105.4 | 81.7 | 174.4 | 0.8 |
| IL-6, pg/ml (IQR) | 6.7 | 3.4 | 8.6 | 4.3 | 2.3 | 7.6 | 0.3 |
| IL-8, pg/ml (IQR) | 34.9 | 32.8 | 52.7 | 37.5 | 32.8 | 63.2 | 0.6 |
| IL-10, pg/ml (IQR) | 3.9 | 1.9 | 8.4 | 5.8 | 2.4 | 9.4 | 0.6 |
| IL-12p70, pg/ml (IQR) | 3.4 | 1.4 | 4.6 | 3.4 | 1.4 | 5.2 | 0.9 |
| IL-17A, pg/ml (IQR) | 0.2 | 0.2 | 0.3 | 0.2 | 0.2 | 0.5 | 0.6 |
| IL-18, pg/ml (IQR) | 254.4 | 212.5 | 563.5 | 279.5 | 231.5 | 482.1 | 0.8 |
| IL-23, pg/ml (IQR) | 3.2 | 3.2 | 4.8 | 3.2 | 3.2 | 5.6 | 0.8 |
| IL-33, pg/ml (IQR) | 15.5 | 9.0 | 32.8 | 15.9 | 7.2 | 57.1 | 0.9 |
|  | **Decompensated cirrhosis** | | | | | |  |
|  | **Jugular veins** | **IQR** | | **Hepatic veins** | **IQR** | |  |
| IL-1β, pg/ml (IQR) | 15.3 | 15.3 | 48.5 | 15.3 | 15.3 | 34.3 | 1 |
| IFN-α2, pg/ml (IQR) | 3.5 | 1.5 | 6.1 | 3.9 | 1.3 | 6.9 | 0.8 |
| IFN-γ, pg/ml (IQR) | 12.9 | 3.1 | 21.6 | 11.5 | 3.5 | 23.8 | 1 |
| TNF-α, pg/ml (IQR) | 13.3 | 2.9 | 32.5 | 17.7 | 1.8 | 34.7 | 0.8 |
| MCP-1, pg/ml (IQR) | 114.0 | 70.0 | 206.0 | 116.3 | 76.2 | 232.6 | 0.9 |
| IL-6, pg/ml (IQR) | 22.5 | 11.6 | 45.5 | 20.7 | 11.7 | 45.5 | 0.6 |
| IL-8, pg/ml (IQR) | 78.0 | 41.9 | 260.8 | 114.2 | 38.0 | 293.8 | 0.6 |
| IL-10, pg/ml (IQR) | 20.4 | 8.3 | 44.2 | 19.3 | 8.1 | 50.2 | 0.8 |
| IL-12p70, pg/ml (IQR) | 5.0 | 1.5 | 16.5 | 4.4 | 1.5 | 19.4 | 0.8 |
| IL-17A, pg/ml (IQR) | 0.8 | 0.2 | 1.7 | 0.5 | 0.2 | 1.9 | 0.9 |
| IL-18, pg/ml (IQR) | 388.2 | 272.3 | 767.9 | 414.4 | 293.9 | 675.5 | 1 |
| IL-23, pg/ml (IQR) | 19.1 | 3.2 | 31.3 | 18.2 | 3.2 | 50.9 | 0.6 |
| IL-33, pg/ml (IQR) | 61.5 | 12.3 | 172.5 | 63.2 | 18.5 | 207.1 | 0.8 |

**Supplementary table 4.** Comparison of C-reactive protein concentration between patients with and without CSPH in blood from jugular veins.

|  | **Jugular veins** | | | | | |  |
| --- | --- | --- | --- | --- | --- | --- | --- |
|  | **Non-CSPH** | **IQR** | | **CSPH** | **IQR** | | **p-values** |
| CRP, mg/l (IQR) | 4.2 | 2.5 | 10.7 | 7.6 | 5.0 | 20.3 | 0.1 |

Data are expressed as median with interquartile ranges (IQR). P-values were assessed by Mann-Whitney-U-Test. CSPH, Clinical significant portal hypertension; CRP, c-reactive protein.

**Supplementary table 5.** Comparison of C-reactive protein concentration between patients with compensated and decompensated cirrhosis in blood from jugular veins.

|  | **Jugular veins** | | | | | |  |
| --- | --- | --- | --- | --- | --- | --- | --- |
|  | **Compensated** | **IQR** | | **Decompensated** | **IQR** | | **p-values** |
| CRP, mg/l (IQR) | 4.1 | 2.4 | 6.0 | 11.0 | 6.0 | 21.0 | 0.009 |

Data are expressed as median with interquartile ranges (IQR). P-values were assessed by Mann-Whitney-U-Test. When significance between the groups was reached, values are printed in bold. CRP, c-reactive protein.

**
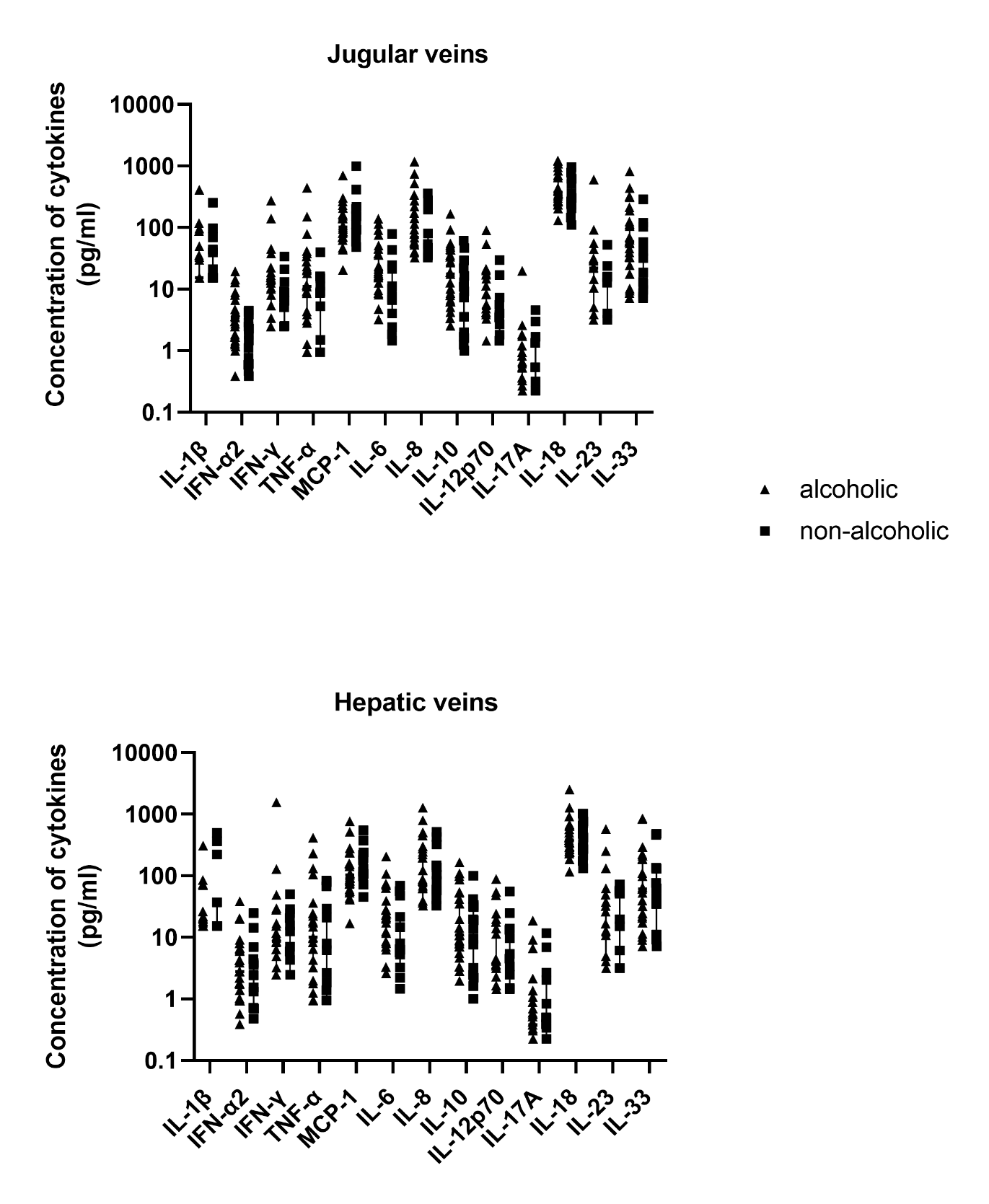
**

**Supplementary figure 1.** Comparison of serum cytokine levels between blood from hepatic and jugular veins in patients with alcoholic versus non-alcoholic cirrhosis.
